# Type and Context of Alcohol-Related Injury among Patients Presenting to Emergency Departments in a Caribbean Country

**DOI:** 10.3390/ijerph14080877

**Published:** 2017-08-04

**Authors:** Sandra D. Reid, Jannel Gentius

**Affiliations:** 1 Department of Psychiatry, The University of the West Indies, St. Augustine, Trinidad and Tobago; 2 Caribbean Institute on Addictive Disorders, Petit Bourg, Trinidad and Tobago; 3 Department of Behavioural Sciences, The University of the West Indies, St. Augustine, Trinidad and Tobago; jannelphilip@yahoo.com

**Keywords:** alcohol, injury, emergency department, pedestrian, non-driver, Caribbean, Trinidad and Tobago, alcohol outlet density

## Abstract

There is an association between alcohol consumption and injuries in Latin America and the Caribbean. This cross-sectional study explores the socio-contextual factors of alcohol-related injuries in Trinidad and Tobago. Data on drinking patterns, injury type, drinking context prior to injury, and demographics were collected from patients presenting with injuries to the Emergency Departments (ED) of four hospitals. Findings show that 20.6% of patients had consumed alcohol, mainly beer, in the 6 h before injury. More than half were drinking at home (27%), or someone else’s home (27%). Injury most commonly occurred outdoors (36%) while in transit. Alcohol-related injuries occurred mainly because of falling or tripping (31.7%); these patients recorded the highest mean alcohol consumption prior to injury. Most persons who fell (50%) did so at home. Findings highlight the previously unreported significant risk of non-drivers sustaining injures through falling and tripping because of heavy alcohol use. Current interventions to reduce alcohol-related injury have focused on drink driving but there is a need for interventions targeting pedestrians and those who drink at home. A comprehensive multi-component approach including secondary prevention interventions in the medical setting, community educational interventions, enforcement of current legislative policies concerning the sale of alcohol, and policy initiatives surrounding road safety and alcohol outlet density should be implemented.

## 1. Introduction

Alcohol consumption, especially heavy drinking, has been linked to suicide, violence and unintentional injuries [1,2]. The World Health Organization (WHO) has described alcohol as a significant risk factor for morbidity, disability and mortality. According to the Pan American Health Organization (PAHO)/WHO Global Status Report on Alcohol and Health (2014), in 2012 5.9% of all global deaths were attributable to alcohol and, of these, 25.8% were due to intentional and non-intentional injuries, especially the latter (17.1%) [3]. The association between alcohol consumption and injuries has been reported in Latin America and the Caribbean, regions where alcohol use is an accepted part of the culture, alcohol consumption is higher than the world average, and heavy episodic drinking is common [4,5,6,7]. It has been estimated that in 2012, alcohol was responsible for 6.7% of all disability adjusted life years (DALYs) lost in the Americas; about one-third of all alcohol-attributable DALYs in this region are caused by injuries [6]. A clear association has been demonstrated between the average number of drinks consumed and the risk of injury in the Americas [7].

Trinidad and Tobago, a two-island nation, is the southernmost country in the Caribbean. Located just about 11 km from the northeastern coast of Venezuela, Trinidad and Tobago is one of the wealthiest islands in the Caribbean with a gross domestic product of 23.56 billion USD and a gross national income per capita of 17,640 USD in 2015 [8]. The alcohol industry is of important economic value. Trinidad is the larger and more developed of the two islands with an area of 4.828 km^2^, while Tobago occupies 300 km^2^. The majority of the estimated 1.3 million population reside in Trinidad. The culture of Trinidad and Tobago reflects its multi-cultural and ethnic diversity and is characterized by socializing and celebration, and the customary use of alcohol. Liming is a favourite activity throughout the year and describes casual social interactions while sharing alcoholic drinks.

According to the PAHO 2012 report, Health in the Americas [9], Trinidad and Tobago has made significant progress in improving the health of its population, but accidents and injuries continue to be a major contributor to morbidity and mortality. Accidents and injuries, including motor vehicle accidents, have ranked among the top five causes of death since the 1990s and remained among the five leading causes of death in both sexes up to 2012. The report further states that the commonest cause of morbidity requiring medical care in Trinidad and Tobago among all age groups is external causes of injury.

This study was conducted as a part of the World Health Organization Collaborative Study on Alcohol and Injuries [10]. The collaborative study uses the same methodology to gather data on the role and extent of alcohol involvement in non-fatal injuries reported to emergency departments in different countries. The Trinidad and Tobago study assessed the involvement of alcohol in non-fatal injuries among emergency room attendees in four geographical areas in Trinidad and Tobago and explored the contexts in which injury occurred.

Consistent with the WHO Collaborative study, the local study had the following specific objectives:

- To document the proportion of victims of non-fatal injuries with alcohol intoxication in a probability sample of emergency room patients at four hospitals geographically distributed throughout Trinidad and Tobago.
- To examine the context in which drinking had occurred prior to the injury, and other drinking variables.
- To collect information on the association of patterns of drinking with injuries.

## 2. Materials and Methods

The study followed the protocol of the WHO Collaborative Study on Alcohol and Injuries which is described in detail in the report “Prevention of alcohol-related injuries in the Americas: from evidence to policy action” [10]. A cross-sectional study design was used to collect data from eligible patients.

Health care services in Trinidad and Tobago are delivered by five Regional Health Authorities (RHAs), commissioned by the Ministry of Health to manage and provide services in specific municipalities which are geographically determined. The study was conducted at the four general public hospitals in 4 of the 5 RHAs. These are the four largest public hospitals in Trinidad and Tobago:

- Port-of-Spain General Hospital (POSGH) in the North West Regional Health Authority,
- Eric Williams Medical Sciences Complex (EWMSC) in the North Central Regional Health Authority,
- San Fernando General Hospital (SFGH) in the South West Regional Health Authority, and
- Scarborough General Hospital (SGH) in the Tobago Regional Health Authority.

Ethical review and approval for the study were obtained from the Pan American World Health Organization Ethics Review Committee (PAHOERC Ref. No: PAHO-2015-04-0019, approved 29 May 2015), the University of the West Indies Ethics Committee, and the Ethics Committees of the North Central, North West, South West and Tobago Regional Health Authorities. The study was conducted from May to October 2015. The standardized questionnaire from the parent study was reviewed by the research team and culturally adapted for language and types of alcoholic beverages. Twelve university-qualified field workers, from Trinidad and Tobago, with prior research experience were trained on the protocol for data collection (administration of the questionnaire and the ALCO-SENSOR III breathalyzer). The training also served as a pilot for the culturally adapted questionnaire. Following the training, minor changes were made to the flow of the questionnaire. There were no difficulties with the administration of the questionnaire. At each hospital, the logistics of how the data would be collected were determined with senior medical and nursing staff of the emergency department. Once confirmed, logistical flow charts were created to assist field interviewers.

An administered questionnaire collected data on the type, cause, place and context of injury; duration, timing, quantity and location of alcohol use prior to injury; the context in which drinking had occurred prior to the injury; drinking patterns in the prior twenty-four hours, one week and twelve months prior to injury; and patient background information on education, employment, monthly income, residence and ethnicity. Alcohol consumption was validated by breath analysis for alcohol using the ALCO-SENSOR III breathalyzer. The interview averaged twenty-five minutes. Eligible patients gave informed consent before data was collected.

The inclusion and exclusion criteria followed the stipulations of the parent protocol. Study participants were persons presenting to the emergency department because of an accident or injury, including self-inflicted injuries, who:

- were at least 18 years old at the time of the study;
- had given informed consent (unconscious and ventilated patients were not included);
- presented to the ED within 6 h of their injury;
- presented to the ED for the first time for that accident/injury.

Data analysis was conducted using the PASW Statistics version 19 (IBM Corp, Armonk, NY, USA). Descriptive statistics in the form of frequencies, cross-tabulations, and mean comparisons were computed for the variables of interest. Data were analyzed using these descriptive statistics and was guided by the specific objectives of the study. Wilcoxon Signed-Ranks Test, and Chi Square tests of independence were also conducted to test mean differences, and equality in frequency distributions, respectively. Due to the few numbers of women in the study, no gender analysis was undertaken.

## 3. Results

A total of 329 patients met the criteria to be eligible for the study. Of these, 237 consented to the interview, a completion rate of 72%. The completion rate varied by hospital. Patients at the Eric Williams Medical Sciences Complex and the San Fernando General Hospital were more likely to respond to the questionnaire (Table 1).

### 3.1. Proportion of Patients Drinking Alcohol before Their Injuries

Of the 228 persons who answered the question (non-response = 9), 47 respondents (20.6%) indicated that they had consumed alcohol within 6 h before their injury or accident. The following analyses were conducted with data from the 47 respondents who consumed alcohol before the injury. The most commonly consumed beverage was beer. The mean total absolute alcohol for those who drank beer (~5%) in the 6 h before their injury was 76.71 mL SD (84.59), with a range from 14 mL to 336 mL. The most frequently occurring absolute totals were 42 mL (17% of patients) and 84 mL (17% of patients); 10/24 (41.7%) had consumed more than 60 mL of alcohol in the 6 h before their injury. 63.8% of these patients submitted to breath analysis. Among the 30 persons who were breathalysed, the mean blood alcohol concentration suggested by breath analysis was 0.07 g/100 mL with a SD (0.08). The minimum level was 0.0 and the maximum was 0.32 g/100 mL. Most persons (40%) recorded a breathalyzer level of 0.0, while 36.6% recorded breathalyzer levels over 0.08 g/100 mL, the legislated legal limit for driving. The most common reason given for refusal of breath analysis related to concerns about legal consequences.

### 3.2. Description of Patients Who Consumed Alcohol Just before Their Injury

**Figure 1:**
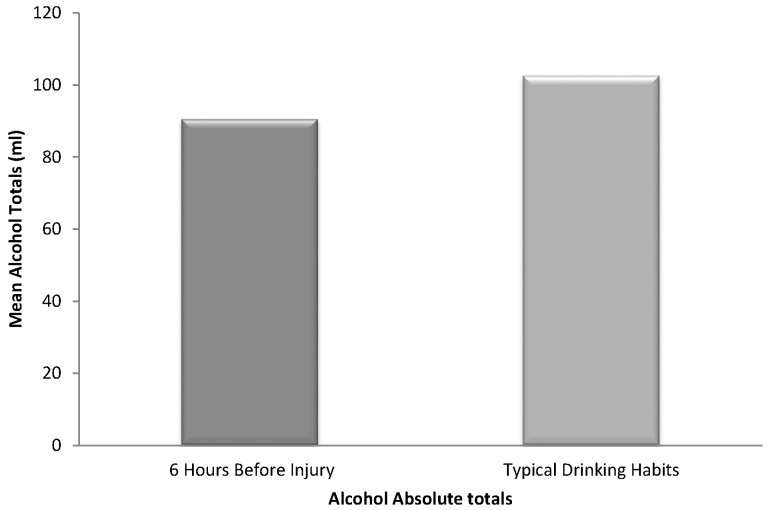
Absolute alcohol totals of typical drinking habits and 6 h before injury.

The patients who drank within 6 h before their injury were mainly male (*n* = 39, 83%). The average age of these patients was 34 years SD (12.68), with a range from 18 years to 61 years. Male patients who indicated that they had alcohol to drink at least 6 h before their injury were most likely to be in the 20–29-year age range (37.8% of males) and females were most likely between 30 and 39 years old (37.5%). The patients who drank within 6 h before their injury or accidents were mainly of African descent (41%), 37% were of East Indian descent, and 20% were of mixed ethnicity. Persons of East Indian and mixed ethnicities tended to be younger (20–29 years) than persons of African descent (40–49 years). The mean number of years of formal schooling was 10 years SD (3.60) but most persons (27%) attended formal education for about 12 years. The majority (74%) were employed and worked at least 30 h per week. Only 47% of patients gave information on their personal monthly income. Among patients who had alcohol-associated injuries, the mean amount of absolute alcohol consumed before the injury was 90.6 mL. These patients reported that in the prior 12 months they typically consumed 102.7 mL of absolute alcohol in one sitting (Figure 1).

A Wilcoxon Signed-Ranks Test indicated that amount of alcohol drank six hours before injury was not statistically higher than the amount typically consumed in the prior 12 months (*Z* = −1.03, *p* = 0.31).

### 3.3. Nature of the Injuries among Patients Who Had Prior Consumption of Alcohol

Injuries sustained by persons who had consumed alcohol in the prior 6 h were mainly unintentional (68.1%); 29.8% were intentional by someone else. The small number of females in the sample did not allow for an assessment of gender differences in the type of injury sustained. Most patients (40.4%) stated that they were at the facility because of a cut, bite, penetrating injury or open wound. Orthopaedic injuries of fracture, strain, sprain or dislocation accounted for 27.7% of injury types. Other reasons for presenting to the emergency department are shown in Table 2.

Patients who were at the hospital for cuts, bites, penetrating injuries or open wounds recorded the highest mean alcohol consumption totals (100 mL) in the six or less hours prior to their injury. A Chi-square test of independence was calculated to test whether the distribution of type of injuries was approximately equal for each type of injury (cells with expected frequencies less than five were excluded from the analysis). A significant difference was found among the type of injuries (Χ^2^ (3) = 10.42, *p* = 0.02). Patients were more likely to have penetrating injuries and open wounds than any other type of injury.

**Figure 2:**
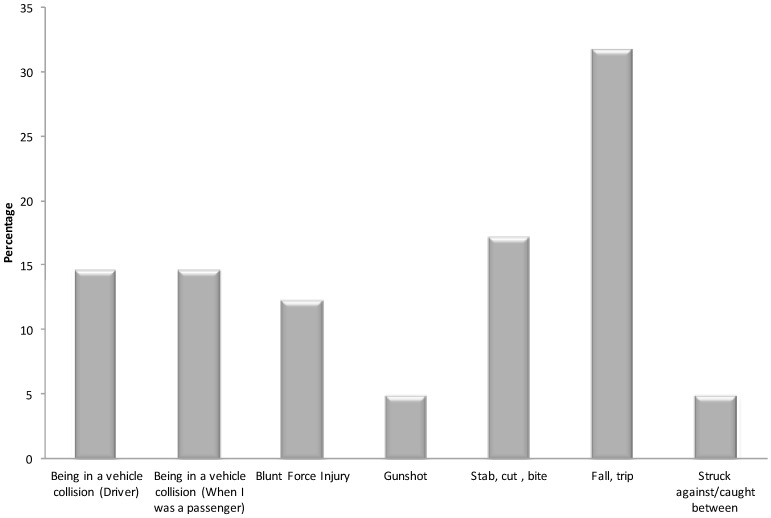
How injury was sustained.

Most patients sustained injury because of falling or tripping (31.7%), or in a motor vehicle collision either as driver or passenger (29.3%). Others indicated that it was due to a stab, cut or bite (17.1%) or blunt force injury (12.2%) (See Figure 2).

A Chi-square test of independence was calculated to test whether the distribution of how patients were injured was approximately the same for each mode of injury (cells with expected frequencies less than five were excluded from the analysis). A significant difference was not found (Χ^2^ (4) = 5.57, *p* = 0.23).

Among the 20–29 age group, (*n* = 15), 33.3% sustained injury by vehicular collision as a passenger, 20% recorded injuries sustained by vehicular collision as a driver and another 20% sustained injuries by falling or tripping; 13% sustained blunt force injury and another 13.3% by stabs, cuts or bites.

Among the 30–39 age group (*n* = 8), most (37.5%) sustained injuries through falling or tripping, 25% by blunt force. Injuries sustained by being struck, stabbed, and being in a vehicular collision when driving, each accounted for 12.5% of patients within this age group.

The majority (50%) of persons in the 40–49 age group (*n* = 6), sustained injuries by falling or tripping. Injuries sustained by vehicular collision as a driver, gunshot, and stabbing, each accounted for 16.7% of patients within this age group.

The majority, (60%), of 50–59-year-olds (*n* = 5), sustained injuries by falling or tripping. Injuries sustained by vehicular collision as a passenger, and stabbing, each accounted for 16.7% of patients within this age group.

A Chi-square test of independence was calculated to test whether the frequency of injuries was approximately the same for each age group (cells with expected frequencies less than five were excluded from the analysis). A significant difference was not found (Χ^2^ (3) = 7.18, *p* = 0.06). Therefore, no one age group was significantly more likely to sustain any injury than the other age groups.

Males (77%) were more likely to have suffered injury by falling than females (23%). Patients who suffered injury by falling or tripping recorded the highest mean amount of absolute alcohol consumed (*n* = 8; M = 126, SD = 132), compared to persons who had blunt force injuries (*n* = 3; M = 65.33, SD = 29.14), and injuries from vehicular collision, (*n* = 4; M = 59.5, SD = 36.2). Among persons who fell, most (50%) were likely to fall at home, than outdoors or at someone else’s home (17%).

### 3.4. Location of Injury

Generally, prior to the accident or injury, patients were just as likely to be in their homes (27%) or drinking at someone else’s home (27%). Sixteen percent (16%) of patients with alcohol-associated injuries were at a pub or some other drinking place prior to their injury or accident. Most patients (36%) indicated that they were at an outdoor public place when the accident occurred, followed by being at home (23%), then in a private vehicle (16%). They tended to be commuting, travelling or walking at the time of their injury or accident (38%); 31% indicated that they were doing leisure activities or playing during their injury/accident. For individuals who drank outside of their homes or at someone else’s home, their injuries occurred outside. None of these individuals incurred injuries in their homes. All persons who were drinking at home also incurred the injury at their homes. As indicated by patients, there was an average of about 2 h and 28 min (148 min) between their last drink and the time of their accident or injury, with the minimum amount of time lapse being 5 min and maximum being 6 h.

## 4. Discussion

In this study, just over one fifth of adults (20.6%) presenting with injuries to the emergency departments of the four largest hospitals in Trinidad and Tobago self-reported the consumption of alcohol within the six hours prior to their injury. This figure is midway within the range of 7–38% reported by other countries globally and in Latin America and the Caribbean [11,12,13,14]. As found in these prior studies, males and younger persons were over-represented among those with alcohol-related injuries. The commonest alcoholic beverage consumed was beer, consistent with previous reports that beer is the most commonly consumed alcoholic beverage among patients with alcohol-injuries [5,15,16], and the most commonly consumed alcoholic beverage in Trinidad and Tobago [9].

The findings of this study support the previously reported role of alcohol as a significant risk factor for accidents and injury presenting to the emergency departments of major hospitals in Trinidad and Tobago [17,18]. The present study is the first to further explore the types of injury and the contexts in which alcohol-injury occurs. Twenty-nine percent (29%) of persons presenting with alcohol-related injuries sustained these as a result of road traffic accidents (RTAs) where they were either the driver or the passenger. Previous research done in Trinidad and Tobago has focused on the role of drink driving in causing morbidity and mortality from RTAs [17,18,19]. Public health interventions have primarily carried a message of “don’t drink and drive” and the major legislative intervention has been the proclamation of the Motor Vehicles and Road Traffic Amendment Act (2007) in 2009 allowing the use of breathalysers. But the commonest cause of injuries associated with alcohol use in this study was the occurrence of accidental falls (31.7%), an association previously described in other populations [20,21].

Both heavy episodic drinking [22,23,24] and acute alcohol intake [2,11,25] are associated with increased risk of alcohol-related injury. Acute alcohol intake is reportedly more predictive of severe injury than pattern of general consumption, but the role of confounders in this association has not been clearly elucidated [2,11,26,27]. The rate of injury is reportedly greatest when both acute and regular use are excessive [27,28]. The World Health Organization defines heavy episodic drinking (HED) as drinking sixty (60) grams or more of pure alcohol on at least one occasion in the past 30 days, and like others, ranks HED as one of the most important indicators for acute consequences of alcohol use including injuries [15,20]. HED is prevalent in Trinidad and Tobago and accepted as a socio-cultural norm. More than half of adult male drinkers (50.4%) and almost one-quarter of adult female drinkers (24.4%) in Trinidad and Tobago engage in heavy episodic drinking [3].

The general pattern of consumption of alcohol among patients presenting with alcohol-related injuries in this study was of chronic heavy drinking. The average consumption of alcohol among patients in this study was 102.7 g in one sitting; and 41.7% reported heavy drinking of more than 60 g of absolute alcohol prior to injury. Greater intake of alcohol had a greater association with falling. Those who were injured by tripping or falling recorded the highest mean alcohol consumption (126 g), suggesting a particularly increased risk of unintentional falls with heavy acute use.

This study also found that among patients presenting to emergency departments in Trinidad and Tobago, drinking mainly occurred in private homes. The home has been previously highlighted as a place where high-risk drinking occurs [29,30,31] and while there is no sanction against excessive drinking at home in Trinidad and Tobago, the impact on safety has not been examined. Reports from other countries have determined the proportion of alcohol-related injuries occurring at home to be between 36% and 39% [29,30,31]. In this study, 23% of alcohol-related injuries were sustained at home. Even though some researchers describe pre-loading, i.e., drinking before going out, it is noteworthy that in this study when drinking occurred at home, the locations of drinking and injury were always the same. Half of the patients who fell after drinking did so at home, consistent with the report of Bunker et al. and others that the most common mechanism for injury at home after drinking was through falls from a low level [29,31].

The finding that most of the alcohol-related injuries in this study (36%) occurred in outdoor public spaces while commuting, travelling or walking supports previous reports of increased risk of injury and death for pedestrians and commuters moving from place to place when under the influence of alcohol [32,33,34]. Dultz et al. found that pedestrians who drink are more likely to cross the street in an unsafe manner and sustain more serious injuries [34]. In Trinidad and Tobago, persons have been educated about drink driving, but walking under the influence of alcohol also increases the risk of injury.

The context of injury related to drinking has not been previously explored in Trinidad and Tobago. The risk of accident and injury posed to non-drivers, specifically pedestrians and persons at home under the influence of alcohol, is a public health problem that is amenable to intervention, and likely contributes significantly to the burden of injury for which care is required at emergency departments. Reports show that pedestrians who had evidence of alcohol use have a higher risk of a road traffic accident, with greater incidence of injuries, and greater risk of fatality than pedestrians who had not consumed alcohol [32,33,34]. Bunker et al. also underscored that alcohol-related injuries sustained at home were associated with more severe and complicated injuries and resulted in more in-patient admissions than those occurring at any other location [31].

The home is a frequent location for drinking in Trinidad and Tobago and while this study suggests a correlation between alcohol use at home and alcohol-related injury especially by falling, until further exploration, the precise role of alcohol in injuries sustained at home remains unknown. Subsequent research should focus on dissecting the multiple factors leading to injury at home, and delineating the role played by alcohol use. This information is needed to guide a variety of public health interventions including the development of policies, prevention strategies and safety guidelines to promote safer use of alcohol in private spaces.

Given the challenge involved in developing interventions to prevent excessive drinking and movement from one place to the next, attention needs to be placed on the walking environment. An important and urgent recommendation from this study, therefore, is for a multi-sectoral approach to similarly explore the public health problem of alcohol-related injuries among pedestrians, particularly those due to falling or tripping. Evaluation, repair and maintenance of transportation infrastructure, including the assurance of safe roads, pathways and sidewalks or crosswalks, as well as traffic safety programmes, and enforcement of traffic laws will increase the safety for pedestrians who have consumed alcohol.

This study supports the accepted report that heavy alcohol use is a major threat to health and safety, and further reports that heavy alcohol use is a significant modifiable risk factor for injury among persons in vehicles, as well as among pedestrians and persons in their homes when they drink. A visit to the emergency department provides an ideal opportunity for the identification of, and intervention for heavy drinking. It is therefore further recommended that all patients presenting with injuries should be assessed for patterns of regular alcohol use and alcohol use before the injury. All patients reporting heavy episodic drinking should receive brief interventions to reduce alcohol intake. Brief screening tools have been shown to be effective, low cost indicators of risky drinking in trauma patients [35,36,37]. The Alcohol Use Disorders Identification Test (AUDIT) was especially found to have a higher sensitivity than blood alcohol levels [35]. These secondary prevention interventions have received mixed results regarding effectiveness in reducing alcohol-related harm [38,39], but most studies suggest reduction in alcohol consumption and/or alcohol-related consequences following various types of brief interventions [40,41,42,43,44,45]. Public health policy initiatives that restrict the availability of alcohol are likely to have even greater impact [46,47].

In Trinidad and Tobago, economic availability of alcohol is controlled through excise and purchase taxes. There are also legislative restrictions through a minimum age to purchase (18 years), a licensing system and limits on hours, days and place of sale of alcohol. While such restrictions are generally associated with a reduction in alcohol consumption and related problems [48], in Trinidad and Tobago they are only partially enforced and relatively toothless. Alcohol is sold on-premise at restaurants, hotels, bars and registered clubs; and off-premise at liquor shops, groceries, supermarkets, convenience stores (including in petrol stations), and small neighbourhood-based shops, through liquor licenses that allow sale at any time of day or night, except during election day when sale and overt display of alcohol are prohibited. Geographic location and density of licensed alcohol outlets impact sale and consumption of alcohol, and prevalence of alcohol-related problems [49,50,51] but globally, restrictions regarding density of retail outlets are much less common than other policy initiatives [52]. Trinidad and Tobago has a high density of alcohol retail outlets. For example, 55% of households in a national survey indicated that there were one to three bars, and 32% had more than three bars within walking distance of the household [53], giving ready access to alcohol. Off-premise outlets are just as densely distributed with implications suggested by the findings of this study, and supported by one study in Australia where a significant majority of alcohol-related attendances to an ED originated from packaged off-premise alcohol sales, consumed in private residences [54].

Based on its findings, this study therefore recommends the increased and consistent enforcement of legislation concerning licensing and retailing of alcohol, and the introduction of new legislative policies to reduce alcohol access, availability and consumption by restricting location and density of retail outlets. This should be augmented by community interventions focused on increasing public awareness of the association between normative alcohol use and the risk of injury. The findings of this study should be re-framed and presented to communities in such a way as to encourage change in the informal cultural rules of drinking and drinking rituals [55].

This study is not without limitations. The reported proportion of alcohol-associated injuries in Trinidad and Tobago is likely a conservative estimate because (1) it does not represent the seasonal patterns of excessive alcohol use that occur in Trinidad and Tobago; (2) the methodology is limiting. The study collected data only from those patients with injuries who met the six-hour arrival criterion. Some patients who presented with injuries and admitted to alcohol use reported delayed help-seeking, coming to the hospital only when pain worsened or when they felt that there was no spontaneous resolution of symptoms. This was particularly so at the Scarborough General Hospital in Tobago. Alcohol can raise the level of the pain threshold, especially when large amounts are consumed [56]. These potentially high alcohol-consuming injured patients would have been excluded from the study as a result. For patients who might have under-reported alcohol use, the high rate of refusal of breathalyzer analysis would likely result in conservative estimates of consumption as well.

Similarly, the study was more likely to miss those patients with more serious injuries, who arrived at the hospital by ambulance and by-passed triage because of the severity of their injuries. Field workers tracked patients referred for hospitalization but were often unable to locate and interview them after they had been admitted elsewhere because of the unavailability of beds on the assigned ward. It has been suggested that patients with more severe injuries are more likely to have used larger quantities of alcohol [11]. Also, with the move towards community-based care, many patients presenting with injuries may have sought medical treatment at the community-based health centres, especially those patients with less severe injuries. Patients of higher socio-economic range similarly may have sought treatment at private medical institutions.

Even among eligible patients, the response rate was low at the hospitals in Scarborough and Port-of-Spain. Because of the demographic distribution in the country, especially around ethnicity, no analysis was therefore attempted on the basis of ethnicity.

## 5. Conclusions

This study presents data that supports the role of alcohol as a significant risk factor for accidents and injury presenting to the emergency departments of major hospitals in Trinidad and Tobago. It highlights the previously unreported significant risk of pedestrians in transit and persons drinking in their homes sustaining injures through falling and tripping as a result of heavy alcohol use. Current interventions related to alcohol and injury have focused on drink driving but there is now a need to further explore the role of alcohol in causing injury among pedestrians and persons who drink at home. These findings should be used to implement interventions targeting pedestrians and to develop multi-sectoral public health interventions which consider the walking environment as well as traffic safety and enforcement of traffic laws. Similarly, there is a need for the development of safety guidelines to promote safe drinking in private spaces, and reduce the risk of injury, especially by falling or tripping, when persons drink at home. Patients presenting to emergency departments in Trinidad and Tobago for injuries should be screened for acute consumption of excessive alcohol and regular heavy episodic use, and appropriate brief interventions and referral should be implemented to ensure safe alcohol use and injury prevention. Enforcement of public health policies related to availability of alcohol, and new initiatives particularly around alcohol outlet density, are also recommended.

## Figures and Tables

**Table 1:** Completion rates among eligible patients at the 4 hospital study sites.

Hospital	No. of Patients Eligible for Interview	No. of Patients Consenting to Be Interviewed	Completion Rate
EWMSC	93	75	80.6%
SGH	24	12	50.0%
SFGH	123	98	79.7%
POSGH	89	52	58.4%
Total	329	237	72.0%

EWMSC: Eric Williams Medical Sciences Complex; SGH: Scarborough General Hospital; SFGH: San Fernando General Hospital; POSGH: Port-of-Spain General Hospital.

**Table 2:** Main reason for presenting to emergency department.

Main Reason for Being Here	Frequency	Percent
Fracture	6	12.8
Strain, sprain, dislocation	7	14.9
Cut, bite, penetrating injury, open wound	19	40.4
Bruise, scrape, superficial wound	9	19.1
Concussion, closed head injury	2	4.3
Organ system injury/multiple organ injury	2	4.3
Other	1	2.1
Unknown	1	2.1
Total	47	100.0
